# An event-related potential examination of contour integration deficits in schizophrenia

**DOI:** 10.3389/fpsyg.2013.00132

**Published:** 2013-03-21

**Authors:** Pamela D. Butler, Ilana Y. Abeles, Steven M. Silverstein, Elisa C. Dias, Nicole G. Weiskopf, Daniel J. Calderone, Pejman Sehatpour

**Affiliations:** ^1^Schizophrenia Research Division, Nathan S. Kline Institute for Psychiatric ResearchOrangeburg, NY, USA; ^2^Department of Psychiatry, NYU School of MedicineNew York, NY, USA; ^3^Department of Psychology, City University of New YorkNew York, NY, USA; ^4^Division of Schizophrenia Research, University of Medicine and Dentistry of New Jersey - University Behavioral HealthCarePiscataway, NJ, USA; ^5^Department of Psychiatry, University of Medicine and Dentistry of New Jersey - Robert Wood Johnson Medical SchoolPiscataway, NJ, USA; ^6^Department of Biomedical Informatics, Columbia UniversityNew York, NY, USA; ^7^Department of Psychiatry, Columbia University College of Physicians and SurgeonsNew York, NY, USA

**Keywords:** schizophrenia, perception, cognition, contour integration, electrophysiology, vision

## Abstract

Perceptual organization, which refers to the ability to integrate fragments of stimuli to form a representation of a whole edge, part, or object, is impaired in schizophrenia. A contour integration paradigm, involving detection of a set of Gabor patches forming an oval contour pointing to the right or left embedded in a field of randomly oriented Gabors, has been developed for use in clinical trials of schizophrenia. The purpose of the present study was to assess contributions of early and later stages of processing to deficits in contour integration, as well as to develop an event-related potential (ERP) analog of this task. Twenty-one patients with schizophrenia and 28 controls participated. The Gabor elements forming the contours were given a low or high degree of orientational jitter, making it either easy or difficult to identify the direction in which the contour was pointing. ERP results showed greater negative peaks at ~165 (N1 component) and ~270 ms for the low-jitter versus the high-jitter contours, with a much greater difference between jitter conditions at 270 ms. This later ERP component was previously termed N_cl_ for closure negativity. Source localization identified the N_cl_ in the lateral occipital object recognition area. Patients showed a significant decrease in the N_cl_, but not N1, compared to controls, and this was associated with impaired behavioral ability to identify contours. In addition, an earlier negative peak was found at ~120 ms (termed N120) that differentiated jitter conditions, had a dorsal stream source, and differed between patients and controls. Patients also showed a deficit in the dorsal stream sensory P1 component. These results are in accord with impairments in distributed circuitry contributing to perceptual organization deficits and provide an ERP analog to the behavioral contour integration task.

## Introduction

Visual integration, also referred to as “perceptual organization,” is impaired in schizophrenia (Silverstein and Keane, 2011). Visual integration is defined as the processes linking the output of neurons, which individually code local (typically small) attributes of a scene, into a global (typically larger) complex structure more suitable for guidance of behavior (Butler et al., 2008). Integration is important for gestalt grouping and object recognition. The importance of visual integration impairments is underscored by inclusion of this domain as a core construct in the Cognitive Neuroscience Treatment Research to Improve Cognition in Schizophrenia (CNTRICS) initiative (Green et al., 2009; Butler et al., 2012; Silverstein et al., 2012).

Visual integration deficits are seen on a number of tasks in schizophrenia including contour integration (Silverstein et al., 2000, 2006, 2012; Uhlhaas and Silverstein, 2005; Kozma-Wiebe et al., 2006; Silverstein and Keane, 2011), coherent motion (Chen, 2011), object recognition from fragmented line drawings (Doniger et al., 2002; Sehatpour et al., 2010), grouping according to proximity or color similarity (Kurylo et al., 2007), and configural processing of faces (Silverstein et al., 2010). A salient aspect of these studies is that they do not appear to be due to a “general deficit,” in that patients perform more accurately than controls when the task relies on judgments about individual features or when grouping interferes with isolating or processing single features (Place and Gilmore, 1980; Silverstein and Keane, 2011).

**Figure 1:**
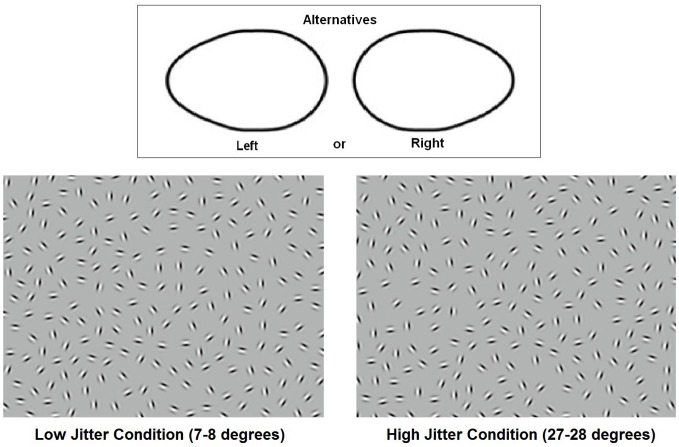
**Stimuli.** The top panel shows the two basic shapes that the participants discriminated. The bottom two panels show examples of low- and high-jitter stimuli.

The present paper focuses on a visual integration paradigm that has been widely used in schizophrenia—contour integration (Field et al., 1993; Silverstein et al., 2000, 2006, 2012; Uhlhaas and Silverstein, 2005; Kozma-Wiebe et al., 2006; Silverstein and Keane, 2011). This task involves viewing Gabor patches that are arranged to form an oval that points to the right or left within a field of randomly-oriented noise Gabor patches (Figure 1). The Gabor signals roughly model the receptive field properties of cells in the primary visual cortex (V1). Importantly, the embedded figure cannot be detected by purely local filters or by neurons with large receptive fields corresponding to the size of the contour (Dakin and Hess, 1998). Thus, the contours are thought to be detected by relying on long-range connections and/or reentrant feedback from V2 or higher areas to produce grouping, and to enhance representation of global shape, especially in the presence of noise (Silverstein and Keane, 2011). There is also some, albeit more inconsistent, evidence for bottom-up contributions to perceptual integration deficits in schizophrenia from studies of contour linking over short distances (Spencer et al., 2003; Keri et al., 2005). Indeed, an fMRI study of contour integration found impairment in a distributed network that includes occipital areas as well as prefrontal, parietal, and ventral temporal areas in patients with schizophrenia (Silverstein et al., 2009).

There is, however, a paucity of information regarding the contributions of early and later stages of visual processing to contour integration in controls and to their impairments in patients with schizophrenia. Event-related potentials (ERPs) are ideally suited to assess different stages of processing due to their high temporal resolution. Previous ERP studies have looked at visual integration in other paradigms such as perceptual closure, which involves identification of fragmented objects (Doniger et al., 2002; Sehatpour et al., 2010). Patients with schizophrenia showed impairment of early-stage sensory processing in this task, as seen by decreased amplitude of dorsal visual stream P1, which occurs at ~100 ms. There is extensive cross-connectivity between brain regions including dorsal and ventral stream areas (Rosa et al., 2009). Information conveyed by the dorsal stream P1 contributes to later stages of processing in the ventral stream lateral occipital complex (LOC) involved in perceptual closure. The ERP signature of perceptual closure, which has been termed closure negativity (N_cl_), occurs at ~300 ms, is impaired in schizophrenia, and has been found to have a source in LOC (Doniger et al., 2002; Sehatpour et al., 2010).

An ERP study, to our knowledge, has not been previously carried out to examine contour integration as described here. The present study assessed the P1, N1, and the closure potential N_cl_ in a contour integration task. While the P1 and N1 occur close in time to each other, peaking at ~100 and ~170 ms, respectively, they reflect very different processes. The P1 has dorsal and ventral stream extrastriate visual cortex sources (Martinez et al., 1999; Di Russo et al., 2003), whereas the N1 appears to reflect primarily ventral stream sources (Allison et al., 1999; Bentin et al., 1999; Doniger et al., 2000). Though additional dorsal stream sources have been demonstrated for the N1 (Sehatpour et al., 2006; Novitskiy et al., 2011), dorsal involvement of N1 is less pronounced than for P1. The P1 is a sensory component, whereas the N1 reflects an initial stage of perceptual processing involving general feature discrimination. For instance, the N1 is larger in response to easy versus difficult to identify shapes, unlike the P1 (Foxe et al., 2005).

Sensory and feature discrimination processes both contribute to later stages of object recognition processing in LOC. This study provides information about contributions of different stages of processing to contour integration impairment in schizophrenia. In addition to assessing these components, a negative component was found that peaked at ~120 ms, which we termed N120. While a behavioral version of this task has recently been optimized and validated for use in clinical trials and fMRI studies as part of the CNTRICS initiative (Silverstein et al., 2012), this study provides an initial exploration of an ERP analog of this task that could be further developed for use in clinical trials.

We hypothesized that the P1 component would not be sensitive to easy versus difficult to identify contours (i.e., low versus high jitter) but that the later components, particularly the N_cl_, would show a differential response to these conditions. In addition, we hypothesized that patients would show a decrease in P1 and N_cl_ components, but not N1, compared to controls.

## Materials and methods

### Participants

Participants consisted of 21 patients meeting Diagnostic and Statistical Manual of Mental Disorder (DSM-IV) criteria for schizophrenia (*n* = 15) or schizoaffective disorder (*n* = 6) and 28 healthy volunteers. Patients were recruited from inpatient and outpatient facilities associated with the Nathan Kline Institute for Psychiatric Research. Diagnoses were obtained using the Structured Clinical Interview for *DSM-IV* (SCID) (First et al., 1997) and all available clinical information. Controls were recruited through the Volunteer Recruitment Pool at the Nathan Kline Institute. Healthy volunteers with a history of SCID-defined Axis I psychiatric disorders were excluded. Patients and controls were excluded if they had any neurological or ophthalmologic disorders that might affect performance or met criteria for alcohol or substance dependence within the last six months or abuse within the last month. All participants had 20/32 or better corrected visual acuity on the Logarithmic Visual Acuity Chart (Precision Vision, LaSalle, IL). This study was approved by the Nathan Kline Institute for Psychiatric Research/Rockland Psychiatric Center and Rockland County Department of Mental Health Institutional Review Board and all participants provided informed consent according to the Declaration of Helsinki.

Groups did not differ significantly in age (patients, 36.8 ± 10.5 y; controls, 35.8 ± 11.8 y; *p* = 0.76) or gender (patients, 20 males, 1 female; controls, 22 males, 6 females, *p* = 0.21). As expected, patients had significantly lower IQ (patients, 92.5 ± 8.1; controls, 105.6 ± 12.9, *p* < 0.001) (Ammons and Ammons, 1962) and education (patients, 11.3 ± 1.4 y; controls, 14.3 ± 2.0 y, *p* < 0.001). Individual IQ data were missing for one patient. Patients were ill for a mean of 13.5 ± 10.2 years and were receiving a mean antipsychotic dose equivalent to 988.59 ± 460.4 mg CPZ per day. Duration of illness data were missing for two patients. Mean Positive and Negative Syndrome Scale (PANSS) (Kay et al., 1987) 5-factor scores (Lindenmayer et al., 1994) for Positive, Negative, and Cognitive (Disorganized) factors were 3.2 (mild to moderate) 2.5 (minimal to mild), and 2.2 (minimal to mild), respectively. PANSS data could not be obtained for one patient.

### Stimuli

Stimuli were obtained from Silverstein and colleagues (Kozma-Wiebe et al., 2006; Silverstein et al., 2009). The carrier spatial frequency of the Gabor patches was 5 cycles/degree and their contrast was approximately 95%. The stimuli consisted of a closed chain of Gabor patches forming an egg-like shape within a background of randomly oriented Gabor stimuli. The spacing between the contour elements was kept constant (8λ; where λ is the wavelength of the Gabor stimulus) as was the average spacing between the background elements. The Δ value (average adjacent background element spacing: contour element spacing) of each image was 0.9. By keeping the signal-to-noise ratio at a constant level below 1.0, participants' performance was a function of the adequacy of long-range interactions between spatial filters; density cues could not be used to detect contours, as is possible with values above 1.0 when contour elements are closer together than background elements. Prior studies indicated that at Δ = 0.9, chronic, state hospitalized schizophrenia patients are able to detect contours accurately in the absence of orientational jitter (Silverstein et al., 2000, 2006). Two orientation levels were used: 7–8° (low jitter) and 27–28° (high jitter) (Figure 1). This resulted in a small misalignment of the contours in the low-jitter condition and a much greater misalignment in the high-jitter condition. There were 40 stimuli for each jitter level, and these were divided evenly between left- and right- pointing egg-shaped ovals, for a total of 80 stimuli. The egg-shaped ovals were always roughly in the center of the image, although each contour varied slightly in size, local and global curvature, and spatial location. Stimuli were presented on a Phillips CRT monitor located 114 cm from the participants. Visual angle was 9.5 × 7 degrees. The mean luminance of the monitor was 65 cd/m^2^.

### Procedure

The 80 stimuli were intermixed randomly and shown for 250 ms each with an interstimulus interval (ISI) of 2000 ms. Participants were instructed to look at the center of the screen, and to press the left or right mouse button if the contour pointed toward the left or right, respectively. Practice trials were given with the low-jitter stimuli in blocks of twenty trials until participants scored above chance on at least one block. During the ERP experiment, all 80 unique stimuli were viewed once in each block, and the blocks were approximately 3 min long. Eight blocks were conducted overall, allowing participants to view 320 trials each of low- and high-jitter stimuli. Participants were periodically prompted to stay focused. The behavioral outcome measure was the percent of correct responses.

### Data acquisition

High-density continuous EEG was acquired from 64 surface electrode sites that were arranged equidistant from each other, using the ANT/Duke layout and EEProbe acquisition system (ANT, Enschede, The Netherlands), along with digital stimulus timing-tags. Data were digitized online at 512 Hz. All data were recorded relative to a common average reference (i.e., average of all electrodes) online. Epochs (−100 to 400 ms) were created off-line. Data were baseline corrected from −100 ms to stimulus onset and an artifact rejection of ±120 μv was applied to all electrodes. Trials containing eye-movements, identified as deflections of >10 μ V lasting >25 ms appearing on both eye channels, were rejected offline. The number of trials that survived artifact rejection for patients was 206 ± 68.9 and for controls was 255 ± 51.8 and was significantly different between groups (*t*_(47)_ = 2.9, *p* = 0.006). Epochs were averaged for each participant for each jitter condition. A spherical spline algorithm was implemented to compute the current source density (CSD) of the EEG. The CSD represents an improvement over more commonly used voltage measures as it decreases the effects of volume conduction and acts as a high-pass spatial filter, which reduces the overlap between ERPs at different sites (Saron et al., 2003; Whitford et al., 2011) and has been used in previous schizophrenia studies (Whitford et al., 2011; Kayser et al., 2012).

### ERP components

CSD topography maps were used to select the electrode sites with most prominent P1, N120, N1, and N_cl_ components. P1 was maximal over dorsal-occipital electrodes (right hemisphere: P4, P6, PO8; left hemisphere: P3, P5, PO7), N120 over central electrodes (P0z and Oz), and N1 and N_cl_ over lateral, ventral-occipital sites (right hemisphere: P8, PO8, PO10; left hemisphere: P7, PO7, and PO9). The mean CSD was obtained for each component over specific latency windows (P1: 85–115 ms; N120: 105–135 ms; N1: 150–180 ms; N_cl_: 250–290 ms). Grand average waveforms were constructed separately for low and high jitter for patients and controls. Figures show filtered waveform data (50 Hz low-pass, 24 dB/octave roll-off).

### Statistical analysis

Between-group analyses for the behavioral measure (percent correct) and each ERP component (P1, N120, N1, or N_cl_) were performed separately using mixed-model analyses of variance (ANOVAs) with group (patient, control) as the between-subjects factor, and with jitter (low, high) and hemisphere (right, left for ERP components) as within-subject factors. Relationships between measures were assessed using Pearson correlation coefficients. Behavioral data were not collected from 1 patient and 2 controls in the low-jitter condition and 2 patients and 2 controls in the high-jitter condition. Effect sizes for ANOVAs are reported as partial eta squared (η^2^_*p*_) to be consistent with past studies of contour integration (Keane et al., 2012; Silverstein et al., 2012).

## Results

### Behavioral results

**Figure 2:**
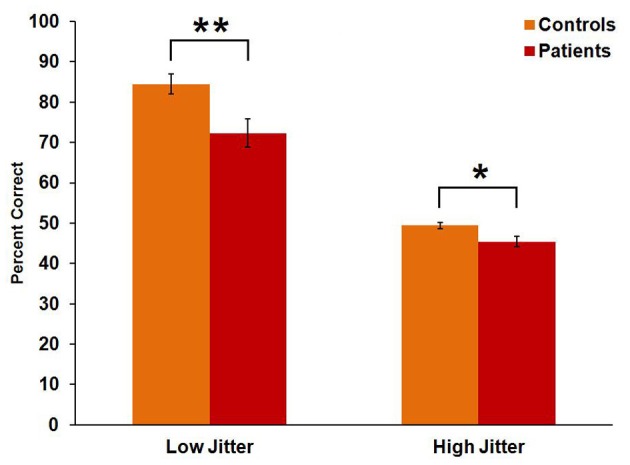
**Behavioral performance of controls and patients in low- and high-jitter conditions.** ^*^p < 0.05, ^**^p < 0.005.

Controls performed significantly better than patients [*F*_(1, 43)_ = 12.7, *p* = 0.001, η^2^_*p*_ = 0.228]. Both groups performed better on the low-jitter condition than on the high-jitter condition [*F*_(1, 43)_ = 198, *p* < 0.001, η^2^_*p*_ = 0.822] (Figure 2). There was also a significant group × jitter interaction [*F*_(1, 43)_ = 4.1, *p* = 0.049, η^2^_*p*_ = 0.087], with a greater between-group difference in the low- than high-jitter condition.

### Electrophysiology results

#### P1

**Figure 3:**
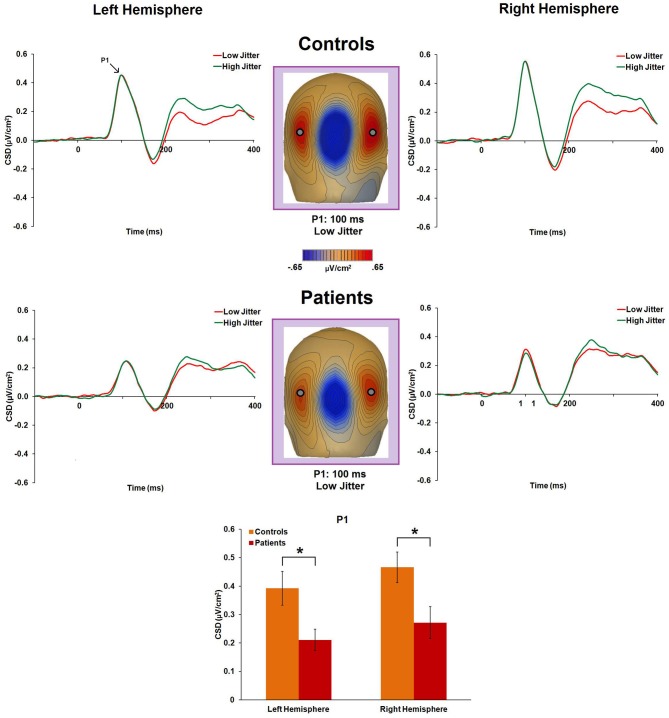
**Event-related potential CSD responses to low- and high-jitter stimuli from occipital electrodes (left hemisphere: P3, P5, PO7; right hemisphere: P4, P6, PO8) for the P1 component.** The graphs indicate no significant differences between the jitter conditions in either group. CSD maps at 100 ms show the observed positivity (P1) in controls and in patients for the low-jitter condition. The bar graph shows significant differences between the groups in the responses to the low-jitter stimuli. ^*^p < 0.05.

P1 CSD was significantly reduced in patients compared to controls [*F*_(1, 47)_ = 8.7, *p* = 0.005, η^2^_*p*_ = 0.156] (Figure 3). Group × jitter [*F*_(1, 47)_ = 1.5, *p* = 0.23, η^2^_*p*_ = 0.031] and group × jitter × hemisphere [*F*_(1, 47)_ = 1.96, *p* = 0.16, η^2^_*p*_ = 0.04] interactions were not significant, reflecting similar P1 CSD to low- and high-jitter stimuli. There was no significant group × hemisphere interaction [*F*_(1, 47)_ = 0.08, *p* = 0.78, η^2^_*p*_ = 0.002] or main effect of hemisphere [*F*_(1, 47)_ = 2.1, *p* = 0.15, η^2^_*p*_ = 0.043].

#### N120

**Figure 4:**
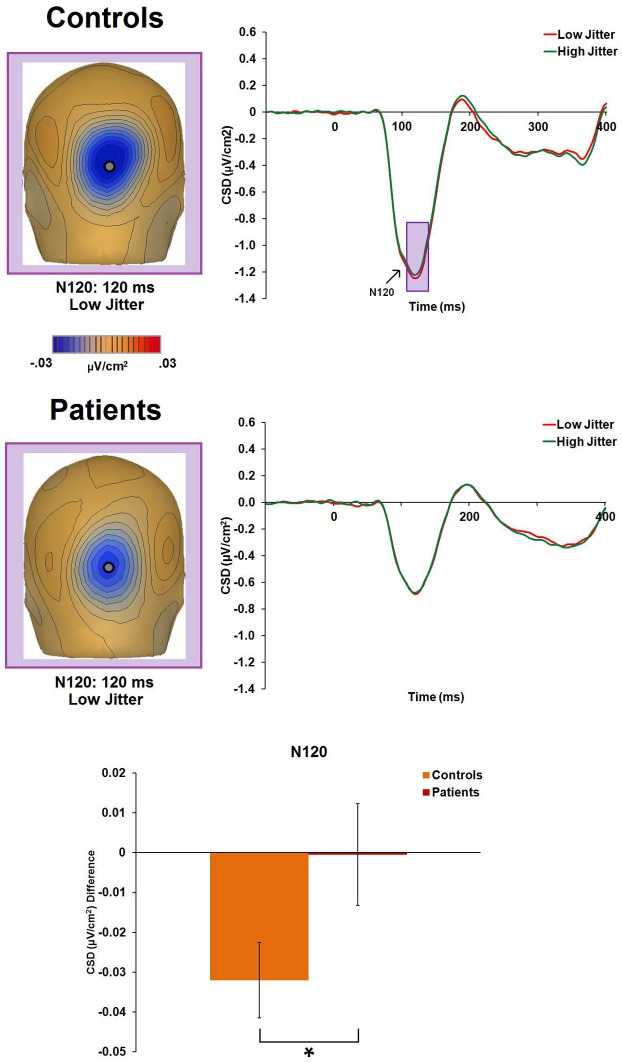
**Event-related potential CSD responses to low- and high-jitter stimuli from occipital electrodes (P0z, Oz) in controls and patients for the N120 component.** The waveforms indicate small but significant differences between the jitter conditions for controls but not for patients. CSD maps at 120 ms show the observed negativity (N120) in controls and patients for the low-jitter condition. The bar graph shows significant differences between the groups in the responses to low- versus high-jitter stimuli. ^*^p < 0.05.

There was a significant main effect of jitter [*F*_(1, 47)_ = 4.4, *p* = 0.04, η^2^_*p*_ = 0.085], indicating differential activity across groups to low- versus high-jitter stimuli (Figure 4). A significant group × jitter interaction was also found [*F*_(1, 47)_ = 4.1, *p* = 0.05, η^2^_*p*_ = 0.081], indicating lack of differential CSD to low versus high jitter in patients [*F*_(1, 20)_ = 0.001, *p* = 0.97, η^2^_*p*_ < 0.001] but differential CSD to the jitter conditions in controls [*F*_(1, 27)_ = 11.5, *p* = 0.002, η^2^_*p*_ = 0.298, a large effect size; (Pallant, 2007)].

#### N1

**Figure 5:**
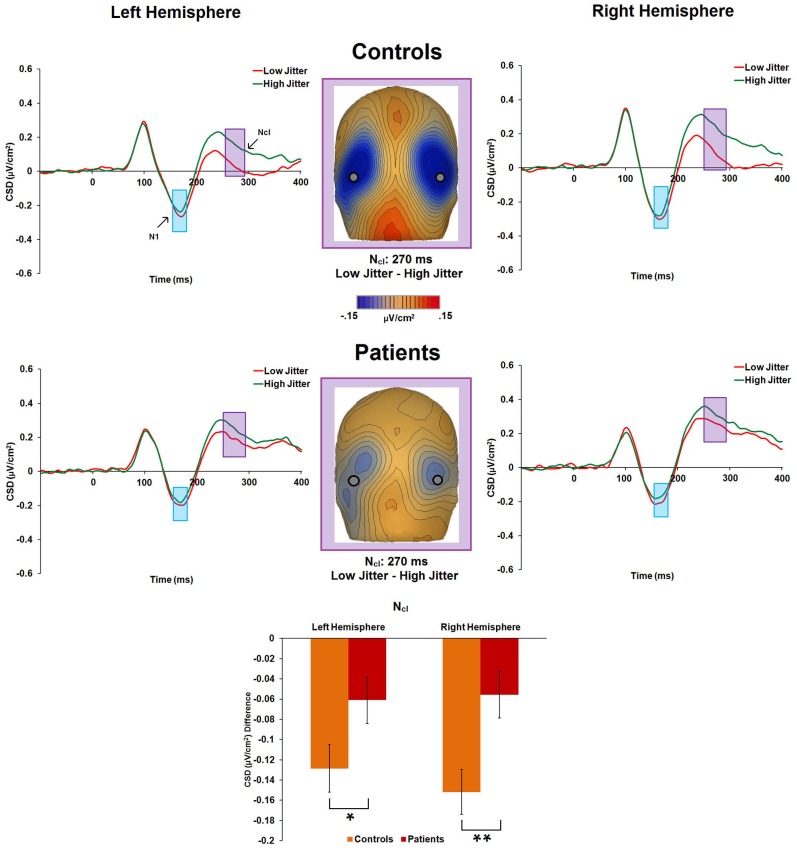
**Event-related potential CSD responses to low- and high-jitter stimuli from occipital electrodes (right hemisphere: P8, PO8, PO10; left hemisphere: P7, PO7, and PO9) in controls and patients.** CSD waveforms show a small difference in response to low- versus high-jitter for the N1 component (blue shaded window) and a much larger difference between conditions for the N_cl_ component (purple shaded window). CSD maps at 270 ms show the observed difference in negativity between the low- and high- jitter conditions for each group in the time-frame of the N_cl_ component. The bar graph shows significant differences between the groups in the responses to low- vs high-jitter stimuli for the N_cl_ component. ^*^p < 0.05, ^**^p < 0.005.

N1 amplitude was not significantly different between groups [*F*_(1, 47)_ = 0.9, *p* = 0.34, η^2^_*p*_ = 0.019] (Figure 5). Group × hemisphere [*F*_(1, 47)_ = 0.29, *p* = 0.59, η^2^_*p*_ = 0.006], group × jitter [*F*_(1, 47)_ = 0.13, *p* = 0.71, η^2^_*p*_ = 0.003], and group × jitter × hemisphere [*F*_(1, 47)_ = 0.21, *p* = 0.65, η^2^_*p*_ = 0.004] interactions were not significant. However, the low-jitter condition produced a more negative peak than the high-jitter condition [*F*_(1, 47)_ = 17.19, *p* < 0.001, η^2^_*p*_ = 0.268].

#### N_cl_

There was a significant main effect of jitter [*F*_(1, 47)_ = 47.8, *p* < 0.001, η^2^_*p*_ = 0.504], indicating differential activity across groups to low- versus high-jitter stimuli (Figure 5). A significant group × jitter interaction was also found [*F*_(1, 47)_ = 8.2, *p* = 0.006, η^2^_*p*_ = 0.148], indicating reduced differential activity to low versus high jitter in patients [*F*_(1, 20)_ = 8.1, *p* = 0.01, η^2^_*p*_ = 0.288] compared to controls [*F*_(1, 27)_ = 51.5, *p* < 0.001, η^2^_*p*_ = 0.656]. Group × hemisphere [*F*_(1, 47)_ = 0.02, *p* = 0.884, η^2^_*p*_ < 0.001], jitter × hemisphere [*F*_(1, 47)_ = 0.29, *p* = 0.59, η^2^_*p*_ = 0.006], and group × jitter × hemisphere [*F*_(1, 47)_ = 0.76, *p* = 0.39, η^2^_*p*_ = 0.016] interactions were not significant.

### Results for participants performing at 70 percent correct or better

To assess whether the ERP results were due to difficulty of patients in identifying the contour and/or to a general deficit, analyses were also carried out with only those participants reaching at least 70% correct in the behavioral low-jitter condition. This resulted in the inclusion of 12 out of 21 patients and 22 out of 28 controls. The general pattern of results was similar to that seen with the entire cohort. For P1, CSD for patients was lower than controls, with a trend for a significant difference between groups [*F*_(1, 32)_ = 3.9, *p* = 0.057, η^2^_*p*_ = 0.109]. For N120, the significant group × jitter interaction [*F*_(1, 32)_ = 4.6, *p* = 0.04, η^2^_*p*_ = 0.127] remained, but the main effect of jitter was no longer significant [*F*_(1, 32)_ = 0.5, *p* = 0.5, η^2^_*p*_ = 0.015]. For N1, the main effect of jitter remained significant [*F*_(1, 32)_ = 12.2, *p* = 0.001, η^2^_*p*_ = 0.276]. For N_cl_, a significant main effect of jitter was still found [*F*_(1, 32)_ = 47.8, *p* < 0.001, η^2^_*p*_ = 0.599] as was a group × jitter interaction [*F*_(1, 32)_ = 4.5, *p* = 0.04, η^2^_*p*_ = 0.123]. This indicates that even when only participants who were clearly performing the low-jitter task above chance were included, there was still a large difference in N_cl_ response to low- versus high-jitter stimuli overall and patients showed a reduced difference between jitter conditions compared to controls. However, while the behavioral deficit was less pronounced than when all participants were included, patients still showed an impaired behavioral response to low- [*t*_(32)_ = 2.1, *p* = 0.045, effect size Cohen's *d* = 0.75 standard deviation units] and high-jitter [*t*_(31)_ = 2.2, *p* = 0.046, effect size Cohen's *d* = 0.95 standard deviation units] conditions compared to controls.

### Source analysis

Inverse dipole modeling was carried out on the grand average waveforms using the brain electrical source algorithm (Scherg, 1990). Models were constructed with three sets of paired dipoles with each set constrained to be symmetrical in location but not orientation, over restricted time intervals corresponding to the components of interest. Dipole locations were calculated based on control data and subsequently applied to patient results. The P1 component was fitted to a window of 85–115 ms accounting for 97% of the variance in controls and 96% in patients. The generator sources were localized to extrastriate Brodmann Area 18 (V2-V3) (Tailarach coordinates: *x*, ±18; *y*, −89; *z*, 13). The N120 component was fitted to a window of 115–135 ms to obtain a clear temporal separation from the P1 component, and accounted for 99% of the variance in controls and 98% of the variance in patients. The generator sources were localized to extrastriate Brodmann Area 18 (Tailarach coordinates: *x*, ±14; *y*, −74; *z*, 16). The N1 component was fitted to a window of 150–180 ms accounting for 96% of the variance in controls and 98% in patients. The N_cl_ was fitted to a window of 250–290 ms accounting for 98% of the variance in controls and 92% in patients. These two components were co-localized to Brodmann Area 37 (fusiform gyrus) (Tailarach coordinates: *x*, ±31.4; *y*, −50; *z*, −13).

### Correlations between ERP measures

ERP components were collapsed across hemisphere because there were no significant main effects or interactions with hemisphere. For P1, correlations were performed for low and high jitter separately. For N120, N1, and N_cl_, high-jitter CSD was subtracted from low-jitter CSD because these components showed a differential response to jitter. P1 responses were significantly correlated with the N_cl_ CSD difference for controls (low jitter: *r* = −0.44, *p* = 0.02; high jitter: *r* = −0.41, *p* = 0.03) but not patients (low jitter: *r* = −0.29, *p* = 0.2; high jitter: *r* = −0.35, *p* = 0.12). In addition, controls showed a trend for (*r* = 0.36, *p* = 0.057) and patients showed a significant (*r* = 0.48, *p* = 0.03) correlation between N1 and N_cl_ CSD differences.

### Correlations between ERP/behavioral and clinical measures

No significant relationships were found between any ERP component or results on the behavioral task and CPZ equivalents or duration of illness for patients. However, using the 5-factor PANSS, patients showed a significant relationship between the Cognitive (Disorganized) factor and both low-jitter behavior (*r* = −0.47, *n* = 19, *p* = 0.04) and N_cl_ CSD (*r* = 0.51, *n* = 20, *p* = 0.02), replicating past findings that poorer contour integration is related to greater disorganization in other domains (Silverstein et al., 2000; Uhlhaas et al., 2006; Silverstein and Keane, 2011). In addition, for patients, there was a significant relationship between percent correct on behavioral performance in the low-jitter condition and N_cl_ (*r* = −0.51, *p* = 0.02), but not other components.

## Discussion

Patients with schizophrenia have impaired visual integration abilities that have been documented in more than 50 studies, spanning over 50 years, as demonstrated by several different research groups, using varied tasks, and in different cultures (Snyder et al., 1961; Izawa and Yamamoto, 2002; Chen et al., 2005; Sehatpour et al., 2010; Silverstein and Keane, 2011). The present study used a contour integration task that was used previously by Silverstein and colleagues (Silverstein et al., 2006, 2009, 2012) to investigate visual integration deficits in schizophrenia. While the contour integration task has been extensively used and optimized for behavioral (Silverstein and Keane, 2011; Silverstein et al., 2012), and fMRI (Silverstein et al., 2009) studies in schizophrenia, there have been no ERP studies that have examined the contributions of early and later stages of processing to these deficits. The present study examined ERP responses to low-jitter stimuli, in which contours were easier to identify, versus high-jitter stimuli, in which contours were more difficult to identify, to further understand contributions of different stages of processing to ability to integrate contours.

As expected, neither patients nor controls showed a differential P1 response to the low- versus high-jitter condition. The P1 component, which peaks at ~100 ms, is an early sensory component and responds to low-level stimulus properties. Aside from the degree of jitter, the low-level stimulus properties of the two conditions were the same and so would not be expected to differentially affect the P1. However, patients showed a significant decrease in the P1 CSD response compared to controls. The current source localization findings of the P1 dipole in extra-striate Brodmann Area 18 are consistent with previous detailed source localization of the dorsal P1 (Di Russo et al., 2001) and with fMRI findings showing reduced activation to Gabor-defined contours in extrastriate visual areas in schizophrenia (Silverstein et al., 2009). The decreased P1 seen in the present study is also consistent with findings from perceptual closure and illusory contour tasks, in which P1 amplitude was similar to scrambled versus unscrambled objects and to recognizable versus non-recognizable illusory shapes, but was reduced in patients versus controls (Foxe et al., 2005; Sehatpour et al., 2010). Decreased P1 has also been seen in a number of studies of schizophrenia (Yeap et al., 2006; Butler et al., 2007; Haenschel et al., 2007; Dias et al., 2011; Martinez et al., 2011), and is thought to reflect impaired magnocellular and/or early-stage cortical processing (Butler et al., 2007; Martinez et al., 2011). Bar and colleagues (Bar, 2003; Kveraga et al., 2007) have suggested that the magnocellular system provides a low-resolution template of stimuli that projects to frontal cortex, which in turn is involved in top-down contributions to object recognition. While a shape rather than an object was shown to participants in the present study, impairment at this early stage of processing, indexed by the P1, may contribute to impaired ability to form a “frame” of the contour.

The N120 component, which peaked at 120 ms, showed a small but significant difference between low- versus high-jitter stimuli. This was unexpected because a component that differentiates between levels of recognizability has not, to our knowledge, been reported earlier than the N1 component. Controls showed a significant effect of jitter whereas for patients, there was an almost total lack of differentiation between the jitter conditions at this point in processing. Thus, this component appears to represent an early cortical process that is sensitive to differing levels of prepotent stimulus organization, and is impaired in patients with schizophrenia. The time-window, orientation, and Tailarach positions of dipoles differed for P1 and N120, though a dorsal stream source was found for both. However, further studies are needed to evaluate this component.

The N1 component, which peaked at ~165 ms, showed a small but significant difference in response to low- versus high-jitter stimuli. This is consistent with previous studies showing a larger N1 when classes of objects rather than presence or absence of objects are required to be detected (Mangun and Hillyard, 1991; Vogel and Luck, 2000) or when illusory contours versus non-contour control stimuli are presented (Murray et al., 2004; Foxe et al., 2005). However, the N1 was not diminished in patients with schizophrenia versus controls. The N1 indexes initial activation of ventral stream object recognition areas (Sehatpour et al., 2010). Thus, while this component appears to be sensitive to jitter conditions, the initial input to the ventral stream appears to be intact, in agreement with previous studies of visual integration in schizophrenia (Doniger et al., 2002; Foxe et al., 2005; Sehatpour et al., 2010).

For controls, the N_cl_ component, peaking at ~270 ms, was highly divergent between the low- and high-jitter conditions, with a much greater negativity seen to the low-jitter than high-jitter stimuli. However, this was not the case for patients, who showed a significantly smaller difference than controls to low- versus high-jitter stimuli. In addition, for patients, better behavioral performance in the low-jitter condition was significantly related to a greater N_cl_ (i.e., a greater difference between low- versus high-jitter CSD), suggesting that this component is important in the actual recognition of contours. The N_cl_, like the N1, was source localized to ventral stream fusiform gyrus, and this region was shown to be involved in contour integration in a prior fMRI study (Silverstein et al., 2009). The intact N1 indicates that rather than intrinsic impairment in this ventral stream area, the impaired N_cl_ response in patients may be due to aberrant input from other brain areas. Indeed, the finding of a significant correlation between P1 and N_cl_ in controls, but not patients, suggests contributions of dorsal stream processes to ventral stream object recognition, which are dysfunctional in patients.

The present ERP results for Gabor-defined contours represent something of a hybrid of results found for perceptual closure, which involves actual objects (Doniger et al., 2002; Sehatpour et al., 2010), and for illusory contours, which involves more automatic shape processing (Murray et al., 2002; Foxe et al., 2005). Similar to the present study, perceptual closure results showed decreased P1 amplitude, a normal N1, and decreased N_cl_ to scrambled versus unscrambled line drawings of objects in patients versus controls (Doniger et al., 2002; Sehatpour et al., 2010). The N_cl_ was localized to ventral stream areas in these studies. A combined fMRI/ERP study of perceptual closure showed that a distributed network was involved in perceptual integration such that impaired activation of dorsal stream visual regions contributed significantly to impaired frontal activation, which in turn contributed to impaired activation of hippocampus and ventral stream regions (Sehatpour et al., 2010). This is consistent with a “frame and fill” model of object recognition (Bar, 2003) in which the low-resolution template generated by the fast magnocellular/dorsal stream provides the “frame” for the fine-detailed information provided by slower parvocellular visual projections to the ventral stream. However, like the illusory contour study (Murray et al., 2004; Foxe et al., 2005), the present study also produced a differential N1 to easy versus difficult to identify stimuli, but did not differ between groups, whereas the objects used in the perceptual closure study produced differential processing only in the later N_cl_ component and not the earlier N1. Thus, both early (i.e., N120, N1) and later (i.e., N_cl_) processes appear to differentiate between recognizability of the Gabor-defined contours.

Using a contour integration task, which was the basis for the task used in the present study, Silverstein and colleagues (Silverstein et al., 2009) found a distributed network of brain activity in controls which included greater recruitment of visual areas V2/V3, and V4 as well as frontal and parietal areas compared to schizophrenia patients in an fMRI study. Silverstein and colleagues suggested that recruitment of anterior areas involved in attention is partly driven by the quality of form representations constructed in the occipital lobe, and, conversely, that extent of activation in the occipital lobe is affected by the amount of feedback from frontal and parietal areas to visual areas to increase the salience of the contours relative to the background noise. They also suggested that the lack of impairment in V1 is consistent with the hypothesis that this area is involved in smaller scale groupings (which can occur normally in schizophrenia). However, in regions progressively anterior to V1, grouping occurs over increasingly larger regions of space (Angelucci et al., 2002), and it is in these regions where processing breaks down in the disorder. Results are consistent with areas found to be activated in controls and non-human primates in contour integration (Kourtzi et al., 2003). While speculative, the present ERP results suggest that the impairment in the contour integration task, as in the perceptual closure task (Sehatpour et al., 2010), may involve impaired input to the frontal cortex from dorsal stream areas and subsequent impaired frontal input to ventral stream areas.

Behavioral performance was impaired in the low-jitter condition for patients versus controls in the present study. In one previous study, performance of patients was not impaired in a low-jitter 7–8° condition (Silverstein et al., 2009), whereas in several other studies it was impaired (Kozma-Wiebe et al., 2006; Silverstein et al., 2012). This may be partly due to prior exposure to a 0-degree jitter condition in some of the studies, but not others (see Silverstein and Keane, 2009; Silverstein et al., 2012). However, it is also likely to be due to differences between studies in stimulus exposure duration; in previous studies duration was 2000 ms whereas it was 250 ms in the present study. This resulted in lower levels of accuracy even for controls in the present study compared to previous studies (Kozma-Wiebe et al., 2006; Silverstein et al., 2009, 2012). Studies using a longer duration stimulus presentation as well as more levels of jitter would be helpful in optimizing the ERP version of this task.

Contour integration deficits were previously found to be related to increased disorganization and/or poor outcome (Uhlhaas et al., 2005, 2006; Silverstein et al., 2005; Silverstein and Keane, 2011). Using the 5-factor PANSS, the present study showed a significant correlation between the Cognitive (Disorganized) factor and low-jitter behavior, in agreement with previous findings, and extended these observations to show a significant relationship between the Cognitive factor and N_cl_ in patients. Indeed, Phillips and Silverstein (2003) suggested that perceptual organization, including contour integration, is a low-level manifestation of a general computational processing deficit involving binding features that are contextually related, which would explain links between impaired perceptual organization and disorganized thinking as well as other cognitive impairments.

It is important to address whether a general deficit and/or impaired motivation or attention could underlie the present results. This does not seem likely for several reasons. While patients performed more poorly than controls on both the low- and high-jitter behavioral conditions, when results were analyzed including only participants who were responding at much greater than chance levels (e.g., 70% correct on the low-jitter condition), patients still showed a significant impairment in N_cl_ compared to controls. In addition, previous studies of contour integration have addressed this issue. For instance, Silverstein and colleagues (Silverstein et al., 2009) found that patients still had decreased activation in areas V2/V3 compared to controls when they were matched to controls on accuracy. Further, as reviewed by Uhlhaas and Silverstein (2005), 10 schizophrenia studies found that patients performed better than controls on tasks in which ability to group stimuli interferes with ability to respond to single stimuli. The superior performance of patients in response to single stimuli is strong evidence for impaired integration independent of a general deficit. Finally, patients did not show a deficit in the N1 component, which would have been expected if results were due to a general deficit.

A further limitation of the study was that all patients were receiving antipsychotic medication. However, CPZ equivalents were not correlated with any ERP component or behavioral performance. In addition, lack of relationship with medication has been found in previous studies of contour integration and a deficit has also been found in un-medicated patients (Silverstein and Keane, 2011). In addition, a limitation is also that this was a relatively small sample size and was almost completely male, particularly in the patient group.

The present results extend previous behavioral and fMRI contour integration findings to the ERP domain. Further, the current contour integration paradigm demonstrated similar ERP signatures as were found in perceptual closure studies (Doniger et al., 2002; Sehatpour et al., 2010) as well as illusory contour studies (Foxe et al., 2005). This may reflect both a general pattern of ERP deficits for perceptual organization and a common impairment in overall perceptual organization in schizophrenia. Taken together, ERP and fMRI data from contour integration, perceptual closure, and illusory contour paradigms complement each other and suggest that impairment in contour integration in patients with schizophrenia occurs at early perceptual levels but also involves higher regions of cortex. This study also provides a pilot assessment of an ERP version of the contour integration task for use in clinical trials.

### Conflict of interest statement

The authors declare that the research was conducted in the absence of any commercial or financial relationships that could be construed as a potential conflict of interest.
